# Pharmacological modulation of adaptive thermogenesis: new clues for obesity management?

**DOI:** 10.1007/s40618-023-02125-0

**Published:** 2023-06-28

**Authors:** V. A. Genchi, G. Palma, G. P. Sorice, R. D’Oria, C. Caccioppoli, N. Marrano, G. Biondi, I. Caruso, A. Cignarelli, A. Natalicchio, L. Laviola, F. Giorgino, S. Perrini

**Affiliations:** Section of Internal Medicine, Endocrinology, Andrology and Metabolic Diseases, Department of Precision and Regenerative Medicine and Ionian Area, University of Bari Aldo Moro, Piazza Giulio Cesare, 11, 70124 Bari, Italy

**Keywords:** Adaptive thermogenesis, Brown adipose tissue, GLP-1R agonists, Incretins, Dual GLP-1/GIP receptor agonists, GLP-1/GCG receptor dual agonists, GLP-1/GIP/glucagon receptor triple agonists

## Abstract

**Background:**

Adaptive thermogenesis represents the main mechanism through which the body generates heat in response to external stimuli, a phenomenon that includes shivering and non-shivering thermogenesis. The non-shivering thermogenesis is mainly exploited by adipose tissue characterized by a brown aspect, which specializes in energy dissipation. A decreased amount of brown adipose tissue has been observed in ageing and chronic illnesses such as obesity, a worldwide health problem characterized by dysfunctional adipose tissue expansion and associated cardiometabolic complications. In the last decades, the discovery of a *trans*-differentiation mechanism (“browning”) within white adipose tissue depots, leading to the generation of brown-like cells, allowed to explore new natural and synthetic compounds able to favour this process and thus enhance thermogenesis with the aim of counteracting obesity. Based on recent findings, brown adipose tissue-activating agents could represent another option in addition to appetite inhibitors and inhibitors of nutrient absorption for obesity treatment.

**Purpose:**

This review investigates the main molecules involved in the physiological (e.g. incretin hormones) and pharmacological (e.g. β3-adrenergic receptors agonists, thyroid receptor agonists, farnesoid X receptor agonists, glucagon-like peptide-1, and glucagon receptor agonists) modulation of adaptive thermogenesis and the signalling mechanisms involved.

## Introduction

Obesity is characterized by a disproportion between caloric intake and energy expenditure (EE) and is frequently associated with high glucose and lipid serum levels [1], as well as by several comorbidities, such as cardiovascular, metabolic, pancreatic, respiratory, oncologic, and sexual diseases [2–5]. In this scenario, the dysfunction of adipose tissue is often accompanied by reduced fat burning due to the lack of activation and reduced amount of brown adipose tissue (BAT) [6]. Indeed, genetic deletion of BAT in vivo results in obesity [7], whereas the stimulation of brown adipocyte differentiation was found to be associated with lower body weight and improved glucose and lipid metabolism [8, 9]. When thermogenesis occurs in BAT, the enhanced EE is mainly fuelled by glucose and fatty acids [10, 11]; therefore, increased BAT activation can significantly improve insulin sensitivity and the lipid profile [11].

The anatomical distribution of BAT in humans has been identified in all ages and in multiple locations, including the cervical, supraclavicular, axillary, abdominal subcutaneous, and paravertebral regions [12–14]. Several studies have highlighted that two distinct types of thermogenic adipocytes exist in mammals: a pre-existing type established during development (the classical brown adipocytes) and an inducible type (the beige or ‘brite’ adipocytes) [15]. The inducible form of thermogenesis, which occurs following exposure to various environmental stimuli such as cold, exercise, or activation of β-adrenoceptors, is marked by the appearance of a cluster of adipocytes within the white adipose tissue (WAT) expressing uncoupling protein 1 (UCP1), a process referred to as ‘browning’ or ‘beigeing’ of WAT [16]. In contrast to brown adipocytes, beige adipocytes emerge postnatally from WAT, even though the origin of these cells is less well understood. In this regard, Min et al. have demonstrated that human beige adipocytes can originate from capillaries in subcutaneous WAT and are responsible for the thermogenic capacity of the white depot when exposed to specific environmental stimuli [17].

Considering this evidence, it has been recently hypothesized that long-term activation of BAT could help to treat obesity and restore a healthy metabolic balance [18]. Therefore, new pharmacological approaches able to convert energy-storing WAT into energy-consuming brown fat may be a potentially effective and harmless solution to obesity, especially in subjects who do not possess appreciable levels of existing BAT.

Currently, most weight-reducing drugs promote the reduction of calorie intake by inducing satiety and fullness (i.e. naltrexone/bupropion) [19] or reducing nutrient absorption (i.e. orlistat) [20]. Nevertheless, these molecules have not shown a significant effect on EE as shown in human studies [21–24]. Indeed, the negative energy balance associated with these approaches does not always warrant to achieve a good weight management in the long term. The decreased EE and reduced adaptive thermogenesis induced by reduced calorie intake represent counter-regulatory mechanisms favouring the fat-loss resistance in obese individuals under these therapies [25]. In addition, reduced adherence to therapy could also lead to weight-loss resistance and weight regain [26]. Therefore, preserving thermogenesis and avoiding the physiological reduction in EE could minimize the risk of weight regain in the long term and reinforce the anti-obesity effect. For this reason, discovering new molecules with thermogenic properties that could drive browning and BAT activation has great potential for obesity management.

Several data showed that BAT activity is subjected to both central and hormonal regulation. Central regulation is effected by the central nervous system (CNS) through efferent sympathetic nerves and is responsive to various stimuli, such as cold temperature [27, 28]. Hormonal regulation depends on the endocrine and paracrine action of different thermogenic signalling molecules, including glucagon and incretins [11, 29–31]. This review focuses on the physiological and pharmacological modulation of adaptive thermogenesis, summarizing the current knowledge on a new class of drugs with broad efficacy in obesity and diabetes. We provide an overview of the thermogenic effects of incretin hormones and their analogues with the aim to explore the main mechanisms whose activation can prove useful in the context of obesity treatment.

## Brown adipose tissue and adaptive thermogenesis

The last decade has brought about increasing interest in the study of BAT, a specialized tissue that mediates energy dissipation by producing heat and maintains body temperature by burning glucose and fatty acids in a process designated as ‘adaptive thermogenesis’ or ‘non-shivering thermogenesis’. Because lipids and glucose represent the key fuel for BAT thermogenesis, it is not surprising that thermogenic BAT activity plays an important role in the regulation of overall energy metabolism and could thus be important for the management of obesity [6]. BAT activity may increase the rate of glucose uptake under insulin stimulation in humans [32, 33]; however, the functions of BAT are limited in the presence of obesity [6, 12, 34–38] and diabetes [6], which may worsen the related metabolic abnormalities. Furthermore, the metabolic derangements associated with obesity (e.g. insulin resistance, adiposity) were restored when BAT was transplanted from metabolically healthy mice to obese mice [39]. Therefore, promoting BAT recruitment and activity has attracted much attention in recent years due to its promising therapeutic potential.

Brown adipocytes originate from the mesodermic compartment during embryonic development. These cells differ from white adipocytes, particularly due to the presence of multilocular vesicles that denote their cellular identity [7]. Brown adipocyte differentiation involves the same transcriptional cascade that is activated for white adipocytes’ commitment, which mainly involves peroxisome proliferator-activated receptor γ (PPARγ) and CCAAT enhancer-binding proteins (CEBPs) [40]. PPARγ is indispensable for the development of all types of adipocytes. CEBPα functions to maintain PPARγ expression, and both cooperatively regulate gene transcription to promote and maintain the differentiated state of adipocytes that can process lipids and glucose and respond to insulin. The absence of CEBPα in mice prevents the development of WAT, but not BAT depots, indicating that lack of CEBPα can be compensated, probably by CEBPβ, during BAT development [41]. Furthermore, specific gene-regulatory networks govern the growth of BAT; the factors involved include PR domain-containing protein 16 (PRDM16) [42] and peroxisome proliferator-activated receptor γ-coactivator-1α (PGC1α) [43]. PRDM16, but not PGC1α, specifically confers brown fat cell identity. PRDM16 binds to and coregulates CEBPβ, PPARγ, PPARα, and PGC1α to promote brown fat-specific gene induction. PGC1α, which also coactivates PPARγ and PPARα, is involved in regulating mitochondrial biogenesis, oxidative metabolism, and thermogenesis.

## Physiological and pharmacological modulation of adaptive thermogenesis

Several studies have investigated the effects of different ligands with thermogenic potential in stimulating BAT activation [44–49]. CNS represents the main transducer of thermogenic responses, whose signals, including adrenergic stimuli and catecholamine [50], fuel sympathetic nerve activity (SNA) in BAT depots under control of specific environmental conditions (e.g. cold) [27, 28]. Other hormones, such as incretins and glucagon (secreted by gut and pancreatic α-cells, respectively) [51–54] appear to sustain BAT activity [29, 30, 55]. Table 1 summarizes current knowledge derived from in vitro, in vivo, and human studies regarding the emerging therapeutic effects of β3-adrenergic receptor agonists, incretin hormones, glucagon, their related pharmacological mimetics, and GLP-1-based multi-agonists, as new treatment options for enhancing thermogenesis and combat obesity.

**Table 1:** Effects of thermogenic drugs in different experimental systems

Thermogenic drugs	Species	In vivo/ex vivo	In vitro	Whole-body
*β3-AR agonist*				
*CL316243*	Mouse	↑ UCP1 *mRNA/*protein in WAT [75]	↑ PKA/p38 MAPK signalling in brown adipocytes [65] ↑ Pgc1α *mRNA* in brown adipocytes [67]	↓ Body weight [74] ↑ EE [76]
*Mirabegron*	Human	↑ ATGL and UCP1 protein in WAT [71] ↑ Beigeing of WAT [83]	–	↑ Insulin sensitivity [11] ↑ HDL serum levels [11] ↑ EE [11] ↓ Glucose levels [83] ↑ BAT glucose uptake [11] ↑ BAT volume [11]
*Rb1*	Mouse	–	↑ AMPK/SIRT1 signalling in 3T3-L1 [71] ↑ UCP1 protein in 3T3-L1 [71]	–
*ESI-09*	Mouse	↑ HSL phosphorylation in WAT [78] ↑ β3-AR expression in WAT [78]	–	↑ EE [78] ↓ Body weight [78] ↑ β3-adrenergic responsiveness [78]
TR agonists				
*Triiodothyronine*	Human	–	↑ UCP1 *mRNA,* ↑ mitochondrial biogenesis in multipotent adipose-derived stem cells [94]	–
	Mouse	–	↑ Mitochondrial autophagy, activity, and turnover in primary brown adipocytes [95]	–
*Levothyroxine*	Human	↑ Glucose uptake in the BAT [105]		↑ EE, ↑ cold-induced thermogenesis [104, 105]
*Liothyronine*	Human	–	–	↑ Skin temperature [107]
*GC-1*	Mouse	↑ Ucp1 *mRNA* in BAT and WAT [96, 102]	↑ UCP1 *mRNA* and protein in white adipocytes [96, 97]	↓ TG serum levels [95] ↑EE, ↓fat mass, ↓ cholesterol [97, 100, 101]
		↑ Multilocular lipid droplet, ↑ UCP1, ↑ mitochondrial DNA content in WAT [97]	↑ Proton-coupled amino acid transporter 2, ↑ Pgc1α, ↑ Prdm16 *mRNA* in mouse preadipocytes [98]	↑ Glucose tolerance, ↑ insulin sensitivity, ↑ body temperature [97] ↓ Body weight [102]
*GC-24*	Mouse	↑ PGC1α and UCP1 protein in BAT [103]	↑ Pgc1β, ↑Pparα, ↑Pparδ, ↑Cpt1, ↑Ucp1 *mRNA* in brown adipocytes [99]	↓ Body weight, ↑ EE [99]
FXR agonists				
*INT-767*	Rabbit	↓ Visceral adipose tissue [114] ↑ Mitochondrial and brown fat-specific markers [114]	↑ UCP1 protein, ↑ mitochondrial biogenesis and function in visceral preadipocytes [114]	↓ Cholesterol, ↓ hepatic steatosis [114]
*BAR502*	Mouse	↑ WAT beigeing/browning [115] ↑ UCP1 protein in WAT [115]	↑ Beigeing in 3T3-L1 preadipocytes [115]	↑ Insulin sensitivity [115]
*Farnesol*	Mouse	↑ AMPKα phosphorylation in BAT [117]	↑ UCP1 protein in 3T3-L1 preadipocytes [116]	–
	Human	–	↑ UCP1 protein in human mesenchymal stem cells [116]	–
*Fexeramine*	Mouse	↑ PGC1α and PGC1β protein in BAT [118] ↑ fatty oxidation, mitochondrial biogenesis in BAT [118]	–	↑EE, ↑ body temperature [118]
*GW4064*	Mouse	↑ lipid droplet size in BAT [120]	–	–
*CDCA*	Mouse	↑ Ucp1 and Pgc1α *mRNA* in BAT [110] ↑ lipid oxidation in WAT [121]	–	↓ Food intake [121]
	Human	↑ BAT activity [126]	–	↑EE [126]
*OCA*	Mouse	↑ BAT activity [123]	–	↓ Body weight, ↓ hepatic steatosis [123]
GLP-1R agonists				
*Exendin 4*	Mouse	↑ UCP1 protein in BAT [149]	↑ PGC1α protein in 3T3-L1 [158] ↑ UCP1 protein in 3T3-L1 [158] ↑ SIRT1 signalling in 3T3-L1 [158]	↓ TG serum levels [149] ↑ BAT glucose uptake [169]
	Human	–	–	↑ EE [30] ↓ Body weight [30]
*Liraglutide*	Mouse	↑ UCP1 protein in WAT [30] ↑ AMPK/SIRT1/PGC1α signalling in WAT [153] ↑ LCAD protein in WAT [155] ↓ ACC2 protein in WAT [155] ↑ sGCβ and PKG1 protein*/mRNA* in WAT [160]	↑ PI3K/AKT/mTOR signalling in C3H10T1/2 cells [143]	↑ EE [145] ↓ Body weight [30, 152] ↓ Food intake [30] ↑ Fatty acid oxidation [155]
	Human	–	–	↑ EE [30] ↓ body weight [30] ↑ BAT glucose uptake [169] –
*Semaglutide*	Mouse	↑ UCP1 protein in WAT [165]	–	↓ Body weight [175]
	Human	–	–	
GLP-1R/GIPR dual agonists				
*Tirzepatide*	Mouse	↑ Ucp1 *mRNA* in BAT [191]	–	↓ Body weight [189] ↑ EE [189] ↑ BCKAs and BCAAs oxidation in BAT [191]
	Human	–	–	↓ Body weight [195]
*NNC0090-2746*	Mouse	–	–	↓ Body weight [188] ↑ EE [188] ↓ Body weight [192]
	Human	–	–	↑ EE [192]
GLP-1R/GCGR dual agonists				
*Aib2 C24 lactam 40 k*	Mouse	↑ phosphorylation of HSL in WAT [215]	–	↓ Body weight [215] ↑ EE [215] ↑ Lipolysis [215]
*DualAG*	Mouse	–	–	↓ Food intake [217] ↓ Glucose levels [217] ↓ Body weight [217]
*Oxyntomodulin*	Human	–	–	↓ Food intake [221] ↓ Body weight [221] ↑ EE [221]
*Cotadutide*	Mouse	↑ Ucp1, Pgc1α, and β3-AR *mRNA* in BAT [219]	–	↓ Glucose levels [217] ↓ Food intake [217]
	Primates	–	–	↑ EE [218] ↓ Body weight [218]
*Mazdutide*	Human	–	–	↓ Body weight [224]
GLP-1R/GIPR/GCGR receptor triple agonists				
*MAR423*	Mouse	–	–	↓ Body weight [226] ↑ EE [226]
*HM15211*	Mouse	–	–	↓ Body weight [47] ↑ EE [47]
*LY3437943*	Mouse	–	–	↓ Body weight [227] ↑ EE [227]
	Human	–	–	↓ Body weight [230]

↑, increase; ↓, decrease; – not available

Acetyl-CoA carboxylase 2 (ACC2), adrenoceptor beta 3 (β3-AR), AMP-activated protein kinase (AMPK), adipose triglyceride lipase (ATGL), brown adipose tissue (BAT), carnitine palmitoyl transferase 1 (CPT1), chenodeoxycholic acid (CDCA), energy expenditure (EE), farnesoid X receptor (FXR), gastric inhibitory polypeptide receptor (GIPR), glucagon-like peptide-1 receptor (GLP-1R), glucagon G-coupled receptor (GCGR), high-density lipoprotein (HDL), hormone-sensitive lipase (HSL), long-chain acyl-CoA dehydrogenase (LCAD), obeticholic acid (OCA), peroxisome proliferator-activated receptor gamma coactivator 1-alpha (PGC1α), proliferator-activated receptor gamma coactivator 1-beta (PGC1β), protein kinase A (PKA), p38α mitogen-activated protein kinase (p38 MAPK), soluble guanylyl cyclase β (sGCβ), protein kinase G 1 (PKG1), sirtuin protein 1 (SIRT1), thyroid receptor (TR), uncoupling protein 1 (UCP1), white adipose tissue (WAT)

### β3-Adrenergic receptor agonists

In the last decade, β3-adrenergic receptor (AR) agonists have emerged as novel pharmacological approaches to counteract obesity and metabolic diseases. ARs are members of the family of G-coupled receptors [56, 57] whose activation is induced by catecholamines [50]. The AR family is composed of three subclasses: α1-ARs, α2-ARs, and β-ARs [58]. Currently, three β-AR subtypes (β1-AR, β2-AR, and β3-AR) have been identified and are encoded by different genes. β3-ARs were identified in several tissues (heart, blood vessels, gallbladder, gastrointestinal tract) and their expression was found to be typically higher in brown depots [59, 60], where non-shivering thermogenesis occurs in response to sympathetic nerve stimulation [27, 28].

**Fig. 1:**
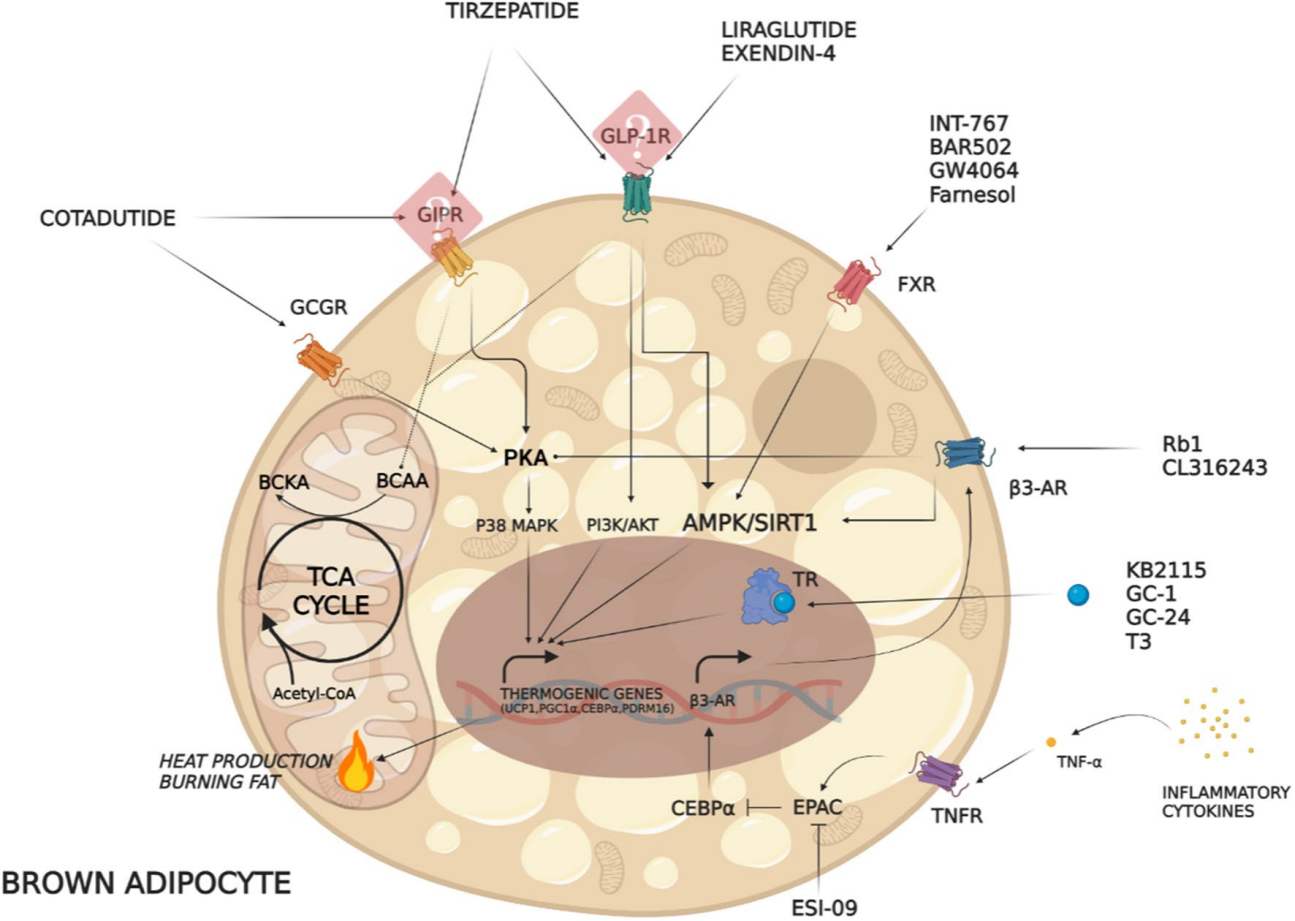
Signalling pathways activated by drugs potentially affecting BAT activity and function: AMP-activated protein kinase (AMPK), adrenoceptor beta 3 (β3-AR), branched-chain amino acids (BCAA), branched-chain keto acids (BCKA), farnesoid X receptor (FXR), gastric inhibitory polypeptide receptor (GIPR), glucagon-like peptide 1 receptor (GLP-1R), Glucagon G-coupled receptor (GCGR), sirtuin-1 (SIRT1), peripheral nervous system (PNS), peroxisome proliferator-activated receptor gamma coactivator 1-alpha (PGC1α), phosphatidylinositol-3 kinase (PI3K), PR domain-containing protein 16 (PRDM16), protein kinase A (PKA), protein kinase B (AKT), p38α mitogen-activated protein kinase (p38 MAPK), ginsenoside Rb1 (Rb1), sympathetic nervous system (SNP), exchange protein directly activated by cAMP (EPAC), EPAC specific inhibitor (ESI-09), CCAAT enhancer-binding protein α (CEBPα), tricarboxylic acid (TCA) cycle, tumour necrosis factor receptor (TNFR), tumour necrosis factor α (TNF-α), thyroid receptor (TR)

β3-ARs are known to exert metabolic effects by enhancing the rate of lipolysis [61] and release of long-chain non-esterified fatty acids (LCFAs) [62, 63] via a Gs-cyclic adenosine monophosphate (cAMP)-protein kinase A (PKA)-dependent pathway (Fig. 1) [64], whose activation also triggers a thermogenic response via upregulation of UCP1 [65]. In this scenario, the activity of the thermogenic protein UCP1, which increases the conductance of the inner mitochondrial membrane to make mitochondria of BAT generate heat rather than ATP, is allowed by an LCFA-dependent mechanism [66]. Furthermore, LCFAs derived from β3-AR-dependent lipolysis act as an energy source to fuel thermogenesis in brown adipocytes [10, 67]. Considering the metabolic actions of the β3-AR–cAMP/PKA axis, researchers have investigated the anti-obesity potential of synthetic ligands that activate these receptors. Several agonists of β3-ARs have been developed, whose effects have been evaluated principally in vivo and in vitro, and to a lesser extent in human studies.

#### In vitro studies

CL316243 was the first adrenergic stimulator whose thermogenic and metabolic activity appears to occur by the activation of PKA–p38 mitogen-activated protein kinase (MAPK) pathway, as observed in mouse brown adipocytes (Fig. 1) [65]. In vitro studies have also clarified the underlying molecular mechanisms mediating the thermogenic response to CL316243, highlighting that this compound caused an increased *mRNA* expression of PGC1α [67], a gene involved in cold-induced thermogenesis and browning [68]. PGC1α requires deacetylation by sirtuin-1 (SIRT1) (Fig. 1) [69], an NAD-dependent protein deacetylase, which acts as a metabolic sensor since its deacetylating activity depends on the intracellular NAD^+^/NADH ratio according to intracellular energetics [70]. The role of β3-adrenergic signalling in regulating thermogenic process was also established in 3T3-L1 adipocytes, in which the incubation with ginsenoside Rb1 (Rb1), a saponin derived from *Panax ginseng*, was shown to act as a β3-AR activator [71]. Rb1 upregulated SIRT1 together with the activation of downstream browning effectors, such as AMP-activated protein kinase α (AMPKα), liver kinase B1, and acetyl-CoA carboxylase (ACC), and increased the rate of lipolysis [71]. Moreover, Rb1 is also known to exert metabolic functions including suppression of triglyceride (TG) accumulation in vitro [72] via activation of AMPK [73], a key factor promoting energy production. These studies suggest that Rb1 can decrease lipid accumulation by inducing the lipolysis–thermogenesis cascade [i.e. AMPKα, adipose triglyceride lipase (ATGL), and UCP1] within WAT depots, confirming its therapeutic potential for counteracting obesity [71]. Overall, these results confirm the key role of PGC1α/SIRT1 signalling in regulating the activity of brown adipose cells induced by the stimulation of β3-AR.

#### In vivo animal models studies

The thermogenic effect of the CL316243 compound was also confirmed by in vivo studies. When infused in obese mice, this molecule increased EE and reduced body weight in association with increased UCP1-dependent thermogenesis in WAT [74, 75]. Notably, CL316243 requires mitochondrial fatty acid oxidation, since obese mice developed a resistance to CL316243-induced thermogenesis when carnitine palmitoyl transferase 2 (CPT2) was genetically abrogated [74]. Furthermore, the efficacy of CL316243 in promoting the activation of brown fat, EE, and weight loss was higher in mice housed at 30 °C compared with mice housed at 22 °C, suggesting that drug efficiency could be also affected by environmental temperature [76]. The efficacy of CL316243 appears also to be reduced in the presence of obesity-associated low-grade inflammation [77, 78], as obese mice with adipose tissue dysfunction and increased proinflammatory cytokines showed impaired β3-AR sensitivity to CL316243, leading to catecholamine resistance and reduced EE [91]. Desensitization of β3-AR occurs through a complex pathway involving exchange protein directly activated by cAMP (EPAC) and Ras-related protein 2 A (RAP2A) [78]. Under physiological conditions, β3-AR expression is regulated by CEBPα, which is a transcriptional factor also involved in the differentiation of new adipose cells as previously discussed [41, 79]. However, in the presence of a proinflammatory milieu, particularly of TNF-α, the EPAC/RAP2A signalling pathway can be activated leading to a cascade of transcriptional events which in turn suppress β3-AR *mRNA* expression through proteasomal degradation of CEBPα in WAT [78, 79]. Beyond promoting the resistance to adrenergic stimuli, EPAC is also known to foster resistance to leptin [80], an adipocyte-derived hormone recently identified as a novel thermolipokine and whose altered signalling may contribute to metabolic dysfunction [81]. Indeed, administration of the EPAC inhibitor ESI-09 in obese mice restored β3-AR expression in WAT, increased EE, and promoted weight loss, thus suggesting that a functional β3-AR signalling is crucial for the maintenance of EE balance. Notably, these benefits were also accompanied with an increased responsiveness to CL316243, together with the activation of hormone-sensitive lipase (HSL) in WAT, a known target of adrenergic signalling [78].

#### Human studies

Mirabegron was the first highly selective β3-AR agonist approved as a therapy against overactive bladder [82]. Beyond this therapeutic indication, recent findings have highlighted a metabolic benefit of this drug, even though few studies have investigated its effects on weight control. O’ Mara et al. observed that when administered daily in young healthy women, mirabegron acutely increased the metabolic activity of BAT, as revealed by the raise of [18F]-2-fluoro-d-2-deoxy-d-glucose uptake, together with the amelioration of EE [11]. The metabolic benefits of mirabegron also extended after 4 weeks of therapy, showing an increase of the amount of BAT in association with the amelioration of lipid profile, insulin sensitivity, and insulin secretion [11]. Noteworthy, these outcomes were more evident in subjects with low amount of BAT at baseline, even though the beneficial responses were not accompanied by changes in body weight and composition [11]. Similarly, Finlin et al. demonstrated that a cohort of older individuals with obesity and insulin resistance treated with mirabegron (50 mg/day for 12 weeks) displayed amelioration of multiple measures of glucose metabolism (i.e. reduced HbA1c levels, increased insulin sensitivity, improved β-cell function) without a significant body weight loss [83]. The same authors also illustrated that these metabolic effects were achieved by the restoration of healthy adipose tissue together with enhanced activation of the beigeing process in subcutaneous WAT depots, an event probably driven by resident macrophages [83].

Considering that the amount of BAT has been generally found to be inversely correlated with BMI [6], stimulation of BAT activation and differentiation with mirabegron may have promising effects in the amelioration of energy balance of obese patients. However, the absence of significant weight-lowering effects observed in obese patients makes this compound less attractive at present. Furthermore, longer-term studies are needed, especially in patients with higher BMI at baseline. Further studies should also elucidate the role of potential catecholamine resistance in the long term in the therapeutic response to mirabegron [78].

### Thyroid receptor agonists

**Fig. 2:**
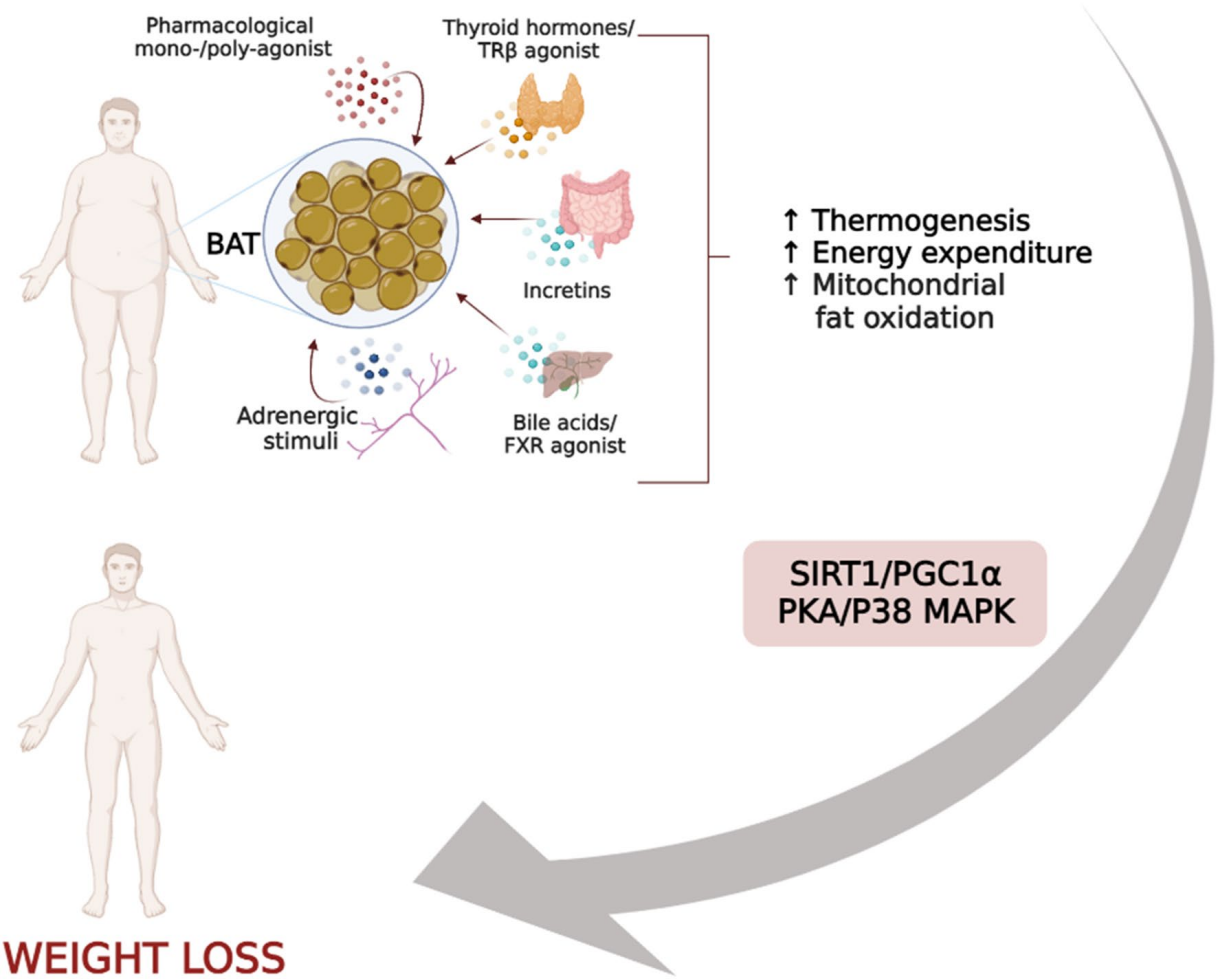
Thermogenic factors may influence BAT activity by inducing SIRT1/PGC1α and PKA/p38 MAPK pathways, thus enhancing EE and promoting weight loss

During the past few decades, growing interest has been directed towards a possible therapeutic use of thyroid hormones (THs) and their mimetics in several dysmetabolic conditions (i.e. dyslipidaemia, liver diseases) and weight management as well, leading to the development of new promising compounds [84, 85]. The effects of THs on metabolic homeostasis were long observed clinically in patients suffering from hyperthyroidism, showing important reductions in body weight, cholesterol levels and, in certain cases, BAT activation [86]. However, the excess of THs is associated with symptoms and signs resulting from increased adrenergic stimulation which may lead to complications, such as tachycardia, atrial fibrillation, osteoporosis, muscle weakness, and anxiety, in the long term [87]. THs were formerly thought to promote their effects on energy homeostasis by directly acting on peripheral tissues, such as BAT and WAT, muscle, heart, and liver [88]. However, several data indicate that THs modulate thermoregulatory control during shivering and non-shivering cold adaptation and food intake, EE, and body weight by acting, to a large extent, at the central level [88, 89]. Indeed, previous findings revealed that central administration of triiodothyronine (T3) in rats resulted in increased EE by stimulating BAT thermogenesis and WAT browning via AMPK-dependent mechanism and upregulation of UCP1 [89] (Figs. 1 and 2). The thermogenic actions of central administration of T3 on BAT and WAT are brought about by suppressing AMPK activity in the ventromedial nucleus of the hypothalamus (VMH). Indeed, genetic ablation of AMPK in the VMH mimics the effects of T3 in the VMH by enhancing BAT thermogenesis and WAT browning [89]. Moreover, the critical role of UCP1 in mediating T3-induced increase in EE has been shown by several studies in which pharmacological or genetic deletion of UCP1 completely blunted the thermogenic action of central T3 [89].

THs activate the specific intracellular receptors (TRs) able to act as transcriptional factors by interacting with coactivators, corepressors, and thyroid response elements on DNA sequences. Two isoforms of TRs have been identified: TRα, mainly located in heart, bone, and brain, and TRβ, principally expressed in tissues with endocrine and metabolic control such as liver and pituitary gland. Notably, both receptors are also implicated in the regulation of thermogenic response, as demonstrated by several animal models where a single or combined knockout of TRα/TRβ resulted in increased diet-induced body weight, impaired glucose tolerance, thermogenesis deficiency and impaired cold adaptation, and reduced the expression of UCP1 in BAT [90, 91]. However, recent findings showed that TRβ is the major isoform mediating T3 actions on multiple metabolic pathways in WAT, including glucose uptake and consumption, de novo lipogenesis, and both UCP1-dependent and -independent thermogenesis [92].

Considering the role of the THs/TRβ system in regulating lipid metabolism, several molecules have been developed with high selectivity for the TRβ isoform including sobetirome (GC-1) and eprotirome (KB2115), which displayed potentially interesting effects on lipids, even though these analogues have shown some unwanted side effects that prevented achievement of the therapeutic market [93]. However, these compounds and other novel analogues (i.e. GC-24) have shown to ameliorate EE and to trigger thermogenic responses when investigated both in vitro and in vivo.

#### In vitro studies

The first evidence of the thermogenic property of THs derive from in vitro studies where human multipotent adipose-derived stem cells treated with T3 for 3 days engaged browning differentiation as shown by the increase of UCP1 *mRNA* expression levels, mitochondrial biogenesis, and oxygen consumption rate [94]. Subsequently, Yau et al. demonstrated that T3-induced thermogenesis occurs via the stimulation of mitochondrial autophagy, activity, and turnover in both a BAT cell line and primary murine brown adipocytes [95]. In view of the thermogenic property of TH, different thyromimetics have been designed with the aim of developing new strategies to induce a BAT-like signature in vitro and for their possible use for the treatment of obesity and its associated metabolic disorders including dyslipidaemia and non-alcoholic fatty liver disease. Ribeiro et al. observed that brown adipocytes isolated from hypothyroid mice treated with the TRβ1-selective thyromimetic, GC-1, showed an increased expression of UCP1, but less activation of adrenergic pathways when compared to brown adipocytes isolated from T3-treated mice, indicating that the stimulation of UCP1 and augmentation of adrenergic responsiveness are mediated by different TR isoforms in the same cells [96]. These effects were better elucidated by Lin and colleagues demonstrating that GC-1 robustly induced UCP1 *mRNA* and protein levels in mouse white adipocytes in a PKA-independent manner when compared to norepinephrine, thus indicating that GC-1-related WAT browning occurs regardless of β-AR activation [97]. The thermogenic power of GC-1 was next confirmed in both bone marrow-derived macrophages and mouse preadipocytes where a significant upregulation of BAT markers (i.e. proton-coupled amino acid transporter 2, PGC1α, PRDM16) after GC-1 administration was observed in an equipotent manner as with T3 stimulation [98]. Similar results have been found in adipocytes from murine BAT that showed increased *mRNA* expression of key thermogenic mediators (i.e. Pgc1β, Pparα, Pparδ, Cpt1, Ucp1, etc.) after chronic treatment with GC-24, a TRβ-selective agonist [99].

#### In vivo animal model studies

The metabolic benefits of TRs agonists were established in animal models of hypothyroidism and hypercholesterolaemia in which the administration of GC-1 resulted in the amelioration of lipid profile and induction of UCP1 *mRNA* expression in BAT, while only minimally mediating synergism between TH and the sympathetic nervous system [96]. Similarly, experiments performed in rodents and primates revealed that both GC-1 and KB-141, another TH analog, favoured the improvement of EE in association with a decrease in fat mass and cholesterol plasma levels [100, 101]. Considering the implication of these molecules in lipid metabolism, more recent preclinical studies examined the anti-obesity and anti-diabetic efficacy of the selective agonist GC-1. GC-1 elicited a remarkable morphological change of subcutaneous WAT in terms of activation of the BAT-like programme of adaptive thermogenesis (i.e. increase of multilocular lipid droplet, UCP1, and mitochondrial DNA content) together with fat loss, metabolic improvement, and recovery of cold tolerance when administered in genetically obese mice [97]. Nevertheless, the browning property of GC-1 appeared to be independent of BAT activity, since this molecule maintained the thermogenic ability in the setting of UCP1 deficiency [97]. The browning of WAT mediated by GC-1 has not been observed by other TR agonists, as reported after KB2115 administration that caused a slight increase in metabolic rate in obese mice without changes in body temperature [97]. Notably, the effects of GC-1 appear to change according to the concentrations used. In fact, obese mice treated with low doses of GC-1 maintained impaired glycaemic control, while higher dose ameliorated glucose tolerance and insulin sensitivity in association with increased body temperature, thus suggesting that the induction of thermogenesis may play a role in mediating these beneficial metabolic effects [97]. The same molecule has also produced favourable results in rat models of type 2 diabetes (T2D) and obesity when administered by a nanochannel membrane device, providing a dramatic body weight reduction, normalization of cholesterol and glucose levels together with sustained expression of UCP1 in WAT [102].

Thereafter, a second generation of TRβ-selective molecules with 40-fold higher affinity compared to TRα has been characterized, GC-24, whose administration for 45 days (8.5 ng/g body weight per day) protected from diet-induced obesity and metabolic alterations via increased resting metabolic response in BAT and upregulation of thermogenic markers (i.e. PGC1α, UCP1) [103]. One year later, Castillo et al. reported that the same GC-24-based therapy (17 ng/g body weight per day) for 36 days accelerated EE by about 15% and limited body weight gain by about 50% in lean mice, effects that were attenuated when this molecule was administered in obese mice [99]. Taken together, these data give support to the awareness that TRβ agonists could eventually become good candidates in the therapeutic arsenal of obesity treatment by mediating the activation of several metabolic responses including thermogenic and browning processes.

#### Human studies

Although both in vivo and in vitro data elucidated the role of THs and their mimetics in regulating BAT activity and browning, few human studies have investigated the effects of TH replacement therapy in this setting. Of note, the thermogenic property of THs was well described in human observational studies regarding hypothyroidism, an endocrine disorder associated with increased cold sensitivity and reduced weight loss following calorie restriction. In this condition, the recovery of euthyroidism through TH replacement therapy (i.e. levothyroxine) was noted to significantly increase cold-induced thermogenesis together with enhanced EE [104, 105]. Similarly, patients with thyroid carcinoma under levothyroxine-based therapy in the post-surgery period showed an enhanced glucose uptake in the BAT during cold exposure [105]. Nevertheless, severe cold intolerance may occur in some hypothyroid patients on levothyroxine therapy with residual symptoms [106]. For this reason, a recent study investigated whether this limitation may be overcome by liothyronine. Indeed, hypothyroid female patients treated with liothyronine showed a reduction of the drop in skin temperature during cold stimulation in both supraclavicular and sternal areas and increased body temperature, thus suggesting that despite restoring the euthyroid condition, levothyroxine therapy exhibited abnormal dermal heat loss and attenuated BAT activation as a sign of deficient T3 receptor action [107].

### Farnesoid X receptor agonists

Farnesoid X receptor (FXR) is a bile acid-activated nuclear receptor, mainly expressed in the liver and intestine playing a major role in regulating bile acids and lipid metabolism. Different clinical trials have reported the effectiveness of FXR modulators in chronic liver diseases and dysmetabolic conditions (i.e. obesity, metabolic syndrome, lipodystrophy, etc.) [108]. Recent findings have also described that FXR may exert actions in extrahepatic tissues such as the kidney, adrenal glands, vascular walls, and adipose tissues. The metabolic role of FXR was better clarified in mice in which whole-body genetic abrogation of this receptor induced glucose and insulin intolerance, together with impaired adipogenesis in WAT and severe cold intolerance [109–111]. Conversely, when FXR knockout mice were exposed to energy overload, a protection from diet-induced obesity and attenuated adipose tissue expansion were observed [112]. In addition to these contrasting results, the direct activity of FXR on BAT function remains ambiguous, even though Yang et al. have recently demonstrated that the expression of this receptor markedly decreased upon cold exposure in murine BAT [113]. The same authors also highlighted that the adipose tissue-specific overexpression of FXR induced a pronounced whitening of BAT and downregulation of mitochondrial functions, thus suggesting that FXR could modulate thermogenesis in a tissue-specific manner [113]. Considering the emerging role of FXR in controlling whole-body energy homeostasis, adipogenesis, and BAT function, many attempts have been made to develop new pharmacological compounds targeting FXR.

#### In vitro studies

Among the plethora of FXR ligands developed, INT-767, a semisynthetic bile acid derivative able to activate both FXR and takeda G protein-coupled receptor 5 (TGR5), has been shown to activate different metabolic pathways in vitro. Indeed, rabbit visceral preadipocytes isolated and treated with INT-767 for 10 days showed increased mitochondrial biogenesis and function as well as increased UCP1 protein expression and oxygen consumption via a cAMP/PKA-dependent pathway (Figs. 1 and 2) [114]. These results are consistent with the recent observation that BAR502, another dual FXR/TGR5 agonist, promoted in vitro trans-differentiation of murine 3T3-L1 preadipocytes into a beige phenotype by increasing intracellular cAMP [115]. Corroborating results were also obtained by farnesol, a natural 15-carbon organic compound found in many essential oils, whose activity increased UCP1 protein levels in both 3T3-L1 adipocytes and human mesenchymal stem cells [116], and ultimately was found to induce thermogenesis via activation of AMPK [117].

#### In vivo animal models studies

Fexaramine was the first gut-restricted FXR agonist with numerous metabolic benefits including WAT browning. In fact, fexaramine-based therapy limited diet-induced weight gain together with enhanced EE and body temperature when administered in obese mice [118]. Furthermore, this molecule has been shown to increase energy utilization by BAT and to upregulate the expression of several BAT activators (i.e. PGC1α, PGC-1β), as well as of their target genes involved in thermogenesis, mitochondrial biogenesis and fatty acid oxidation [118]. The same therapy reduced visceral fat depots, enhanced lipolytic rates and promoted WAT browning in obese mice as compared to untreated controls [118]. Conversely, the chronic administration of a synthetic FXR agonist (GW4064) accentuated body weight gain and glucose intolerance in high-fat diet (HFD)-induced obese mice in association with a decreased bile acid biosynthesis and EE. In addition, worsening of the changes in liver and adipose tissue was observed after GW4064 administration [119]. GW4064 appears also to control energy homeostasis by stimulating thermogenesis through the activation of FXR in brain areas as observed in a mouse model. After intracerebroventricular infusion of GW4064, mice showed important histological remodelling of BAT (i.e. increase of lipid droplet size), which occurred via the control of hypothalamic sympathetic tone [120]. However, these responses were absent in mice with FXR deficiency indicating target selectivity of the compound [120].

The modulation of FXR activity was also investigated by using natural thermogenic nutrients such as bile acids. Diet supplementation with chenodeoxycholic acid (CDCA) or the bile acid-binding resin colestimide, which favours bile acids synthesis, in mice showed an anti-obesity efficacy associated with the increase of UCP1 and PGC1α *mRNA* expression levels in BAT depots [110], reduced food intake and increased lipid oxidation in WAT [121]. Similarly, farnesol, an isoprenoid present in essential oils, has been shown to ameliorate obesity and diabetes [122], and ultimately was found to burn fuel energy by triggering thermogenic responses in HFD-induced obese mice [117]. Notably, farnesol also induced thermogenic factors via AMPKα phosphorylation in BAT under cold acclimation (Fig. 1). In this setting, mice also showed weight loss and reduced BAT and WAT depots as compared to untreated mice [117]. Further, in vivo studies have also observed that obeticholic acid (OCA), a bile acid analogue originally developed for treating liver fatty diseases, exerts a browning effect on WAT. When administered in hyperphagic mice, OCA ameliorated body weight and hepatic steatosis by activating endogenous BAT, but without altering fatty acid oxidation [123].

Based on thermogenic control of bile acids, recent studies have investigated the effects of bile acid mimetics in the regulation of BAT function. For instance, a semisynthetic bile acid derivative INT-767, a dual FXR/TGR5, was found to protect against metabolic disorders and to activate thermogenic pathways in vivo. Indeed, HFD rabbits treated with INT-767 showed a reduction of visceral adipose tissue accumulation, hypercholesterolaemia and non-alcoholic steatohepatitis combined with the upregulation of mitochondrial and BAT-specific markers [114]. In agreement with these results, exposure of obese mice to BAR502, a steroidal dual agonist for FXR and TGR5, resulted in a profound restoration of insulin sensitivity and reshaped the WAT morphology together with the transition towards a beige/brown phenotype as indicated by marked increase of UCP1 protein expression [115].

#### Human studies

Nowadays, few studies have investigated the potential role of FXR–bile acids axis in regulating EE and browning in humans. For instance, modifications of circulating bile acids have been shown to correlate with changes in energy and substrate metabolism upon bariatric surgery [124, 125]. Considering the role of bile acids in the control of BAT function, Broeders and colleagues investigated the effects of oral supplementation of CDCA in healthy females and observed an increase of BAT activity under both thermoneutral conditions and mild cold exposure, which was accompanied by a significant increase in EE (∼5–6%) in the basal, resting state [126]. Other candidates able to target FXR have been examined in clinical trials (i.e. OCA, WAY-362450), showing positive results in the management of cholestatic liver diseases and metabolic syndrome [127], but the effects on thermogenesis and browning processes are still unexplored.

### Glucagon-like peptide 1 receptor agonists

Glucagon-like peptide 1 (GLP-1) is an incretin produced by enteroendocrine L-cells in response to nutrient ingestion that has multiple pleiotropic effects, including the stimulation of insulin release from pancreatic β-cells, inhibition of glucagon secretion [51, 52, 128], regulation of food intake by promoting satiety and fullness [129], delay of gastric emptying [130], and control of energy balance [131]. GLP-1 release from the ileum depends on the composition and size of meals and can be impaired in both obesity and T2D [132, 133]. The insulin-secretagogue effects of GLP-1 are mediated by GLP-1 receptors (GLP-1R), which belong to the class B family of 7-transmembrane-spanning, heterotrimeric G protein-coupled receptors expressed in several tissues, including the pancreatic islet, kidney, heart, stomach, intestine, muscle, and central and peripheral nervous systems [134–138]. However, data from a recent single-cell RNA sequencing analysis investigating the whole-body distribution of GLP-1R have revealed no expression of such receptors in both human and mouse WAT [139, 140]. GLP-1 has a short half-life due to its rapid degradation induced by dipeptidyl peptidase 4 (DPP4), leading to the generation of largely inactive forms (GLP-19–36 amide or GLP-19–37) [136, 141]. Therefore, several structurally optimized GLP-1 analogues with improved bioavailability and sustained action have been developed for clinical use. Among these, liraglutide and exendin-4 have been investigated specifically for their effect on EE.

#### In vitro studies

Exenatide, one of the first GLP-1 receptor agonists (GLP-1RA) developed, was identified as exendin-4 in the salivary glands of the Gila monster (*Heloderma suspectum*) and shares 53% amino acid sequence homology with native human GLP-1 [136]. Conversely, liraglutide is a long-acting GLP-1RA that shares 97% sequence identity with human GLP-1 [142]. Although exenatide and liraglutide differ in terms of amino acid sequence homology with native GLP-1, both compounds showed similar thermogenic capabilities in vitro. The molecular machinery mediating the browning effects of GLP-1RA is complex and appears to include not only effectors of the AMPK pathway, but also protein signalling typically involved in cell growth control, such as the phosphoinositide 3-kinase (PI3K)/AKT/mammalian target of rapamycin (mTOR) pathway, whose activation by liraglutide was found to promote the accumulation of multilocular lipid droplets and mitochondrial biogenesis in brown adipocytes in vitro (Fig. 1) [143]. Similar results have also been reported for exendin-4, which increased the expression of PGC1α and UCP1 via activation of SIRT1 when added to 3T3-L1 adipocytes cultures [144]. These results highlight that SIRT1 is a key bioenergetic regulator shared by both β3-AR- and GLP-1R-mediated thermogenic signalling pathways.

#### In vivo animal models studies

The peripheral and central effects of GLP-1 were elucidated in vivo by using GLP-1R-deficient models and long-acting GLP-1RA. Firstly, Heppner et al. demonstrated that GLP-1R-knockout mice fed an HFD exhibited a blunted response to liraglutide in terms of reduced EE and weight loss when exposed to both noradrenaline and cold temperature [145]. These results indicate the potential involvement of endogenous GLP-1R signalling in the maintenance of both adaptive thermogenesis and body weight. EE modification appears to require the activation of GLP-1R signalling in certain brain areas [146]. The GLP-1R is highly expressed in the hypothalamus, particularly in anorexigenic pro-opiomelanocortin (POMC) neurons within the arcuate nucleus (ARN) and in other areas closely associated with BAT activity (i.e. dorsomedial hypothalamus (DMH) and medial preoptic area (MPOA)) [147]. Indeed, rats with GLP-1R knockdown within DMH develop an obesogenic phenotype due to reduced EE and BAT thermogenesis, with a concomitant increase in hepatic steatosis, insulin resistance, and body weight [148]. Central infusion of exendin-4 in mice induced an increase of sympathetic activity targeting BAT, with increased UCP1 protein expression, and improved TG clearance by robustly increasing TG uptake by brown adipocytes, while this disappeared in the presence of obesity [149]. The amelioration of lipid turnover under pharmacological stimulation of central GLP-1R occurred through a shift in energy metabolism from carbohydrates to fatty acids as the prevalent energy source [149], which confirmed previous data showing that chronic exposure to GLP-1 increased the rate of fatty acid oxidation by BAT [150]. Interestingly, in vivo investigations have described a new hypothalamic pathway that, when activated by liraglutide, was found to trigger BAT thermogenesis, and browning of WAT in an AMPK-dependent manner, regardless of nutrient intake [30].

Both peripheral and central administration of GLP‐1RA stimulated neuronal activity at several hypothalamic sites, resulting in activation of satiety factors and EE in diet-induced obese (DIO) mice [150, 151]. A single intracerebroventricular administration of liraglutide reduced food intake and body weight and increased BAT thermogenesis and UCP1 protein expression in WAT [30, 152]. Similarly, mice challenged with a high-fat and high-sugar diet that were exposed peripherally to liraglutide, beyond changes in eating behaviour, displayed a significant increase in BAT activation and brown fat markers in skeletal muscle and activation of adaptive thermogenesis in WAT via the AMPK–SIRT1–PGC1α pathway (Fig. 1) [153]. Consistent with these findings, selective in vivo ablation of GLP-1R in the neuronal circuits of both DMH and VMH nuclei blunted the effect of liraglutide on body weight, in addition to reducing the thermogenic and browning action mediated by liraglutide and exendin-4 [30, 148, 154]; these observations suggest that energy dissipation might contribute to weight loss in response to these agents.

Despite an increasing body of evidence on the ability of incretin mimetics to elicit BAT responses through both direct and indirect mechanisms, to date few studies have explored the molecular machinery of adipocyte browning induced by GLP-1RA. Recently, liraglutide was shown to participate in mitochondrial fat oxidation by modulating two key enzymes downstream of AMPK signalling (i.e. long-chain acyl-CoA dehydrogenase [LCAD] and acetyl-CoA carboxylase-2 [ACC2]), reducing fat accumulation in association with suppression of adipogenesis, particularly in visceral fat regions of HFD diabetic mice [155]. These events were achieved through induction of UCP1 and PRDM16, key mediators of browning, together with activation of the deacetylase SIRT1 [155], whose involvement in browning was noted [156–159]. Furthermore, when GLP-1R were chronically activated by liraglutide in mice, an increase in the number of mitochondria was observed in both subcutaneous and visceral depots in association with the formation of new beige adipocytes through the activation of soluble guanylyl cyclase (sGC) and protein kinase G 1 (PKG1) [160], which appeared to be novel mediators of the browning process in WAT depots [161, 162].

Considering the thermogenic role of both GLP-1RA and β3‐AR agonist, several studies have investigated the effects of combined administration of both compounds on EE and adipose tissue biology. Rats treated by both liraglutide and the β3‐AR agonist CL316243 showed reduced feeding, enhanced thermogenesis in both epididymal WAT and BAT, and decreased weight gain [163]. The extent of signalling activation by both receptors appeared to change in a tissue-dependent manner: rats exhibited a higher phosphorylation rate of PKA in the liver together with enhanced expression of PPARα and PPARγ, and improved lipid trafficking and resting EE after combined therapy [163]; in contrast, PKA was activated to a lesser extent in BAT and WAT in association with an increase of thermogenic factors (e.g. cytochrome c oxidase subunit 4 isoform 1) and decrease of lipogenic mediators (i.e. PPARα, PPARγ) (Fig. 1) [163]. In this model, the combined therapy shifted the energy balance towards oxidative processes, enhancing peripheral pathways promoting fat mass reduction, liver fat content reduction, and an overall improved metabolic profile [163]. On the basis of these preclinical findings, a combined therapy with GLP-1RA and β3-AR agonists could have potential on metabolic outcomes, even though its efficacy in human obesity is warranted.

More recently, new GLP-1RAs, such as semaglutide or supaglutide, have been developed showing similar or even greater benefits in terms of weight loss, induction of EE and browning of WAT in mice models of obesity, thus further corroborating previous evidence regarding the thermogenic role of GLP-1R activation [164, 165]. Taken together, these results suggest that, at least in experimental animal models, the body weight-lowering effect of GLP-1RA, particularly of liraglutide, may occur by increasing EE through a thermogenic mechanism, independent of changes in caloric intake. However, clinical data have not always supported these promising experimental findings, as GLP-1RA-based therapy was found to lower energy intake without affecting EE or adaptive thermogenesis in humans [44].

#### Human studies

Although it was found that body weight reduction induced by GLP-1RA may occur at least partially through increased thermogenesis and EE in experimental animals, conflicting results have been obtained so far in humans. It was reported that long-term exposure to exenatide or liraglutide in individuals with obesity and T2D resulted in a reduction in BMI and fat mass and an increase in EE [30]. Nevertheless, these findings are in contrast with previous results showing that the anti-obesity efficacy of short-term therapy with GLP-1RA was not accompanied by an improvement in EE [166–168]. Indeed, evidence of the thermogenic effect of GLP-1RAs in humans was provided by performing a tomography scanning of healthy volunteers treated with exendin-4 for 12 weeks, showing an increase in BAT glucose uptake [169]. Although effects on EE could not be detected after 12 weeks, they were reported in another study in T2D patients when exendin-4-based therapy was extended at one year [30]. Similar effects were also observed after administration of liraglutide [30, 169], a drug currently used for both T2D and obesity management [170, 171]. Although liraglutide shows an improved bioavailability as compared to native GLP-1, it requires daily injections, thus potentially reducing therapy adherence [142]. The longer acting analogue semaglutide, with once-weekly administration and greater weight-loss efficacy as compared to liraglutide [172], may provide greater clinical benefit. Semaglutide shows similar thermogenic capabilities in experimental animals [165] and appears to be the most effective in reducing weight among the various GLP-1RA, with a mean weight loss of 15.8% after 68 weeks of treatment in obese and overweight patients [173]. However, the potential thermogenic effects of semaglutide have not been yet elucidated in humans. A clinical trial to clarify the role of BAT activation in mediating the weight loss induced by semaglutide in obese patients is being carried out, with results expected to be available in 2023 [174].

### GLP-1R/glucose-dependent insulinotropic polypeptide receptor dual agonists

Glucose-dependent insulinotropic polypeptide (GIP) is another insulinotropic protein secreted by enteroendocrine K cells of the small intestine acute ingestion of dietary nutrients [175]. Evidence from in vivo studies reported conflicting results regarding GIP release in T2D, with some studies showing enhanced [176] or unchanged secretion [133, 134]. The release of GIP is also modified under cold acclimatation, as observed in rats in which chronic cold exposure significantly augmented circulating levels of GIP in association with an increase in BAT mass [177]. GIP acts through the activation of G protein-coupled receptor (GIPR), whose expression has also been observed in extra-pancreatic tissues, including the hypothalamus and adipose tissue, suggesting that it controls systemic metabolism beyond insulin secretion [178]. The distribution of GIPR at the adipose tissue level was already established, particularly in preadipocytes [179], in which GIPR expression levels are enhanced after adipocyte commitment [180]. However, the expression of GIPR in both mouse and human WAT remains questionable, since analysis of single-nucleus RNA sequencing has recently found GIPR to be predominantly localized in nonadipocyte cells with prevalent expression in pericytes and mesothelial cells [139, 140], suggesting that the effects of GIP on adipocytes could be exerted through indirect mechanisms. Notably, GIPR expression levels in adipose tissue were found to be negatively correlated with adiposity and positively associated with insulin sensitivity [181]. In particular, a defective GIP/GIPR signalling has been observed in the subcutaneous WAT of individuals with obesity and insulin resistance [181]. In accordance, we demonstrated that the excess of saturated fatty acids makes pancreatic β cells dysfunctional in terms of insulin release by reducing protein expression of GLP-1R and GIPR by 50% and 30%, respectively [182], thus suggesting that lipotoxicity could impair the incretin cascades also in other cell types.

The role of GIPR signalling in the regulation of both systemic and adipose tissue metabolism is not fully elucidated, since transgenic animal models produced conflicting results. Mantelmacher et al. demonstrated that genetic interruption of the GIP/GIPR axis in myeloid cells favoured weight gain and metabolic abnormalities concomitantly with impaired EE, adipocyte hypertrophy, and reduced expression of thermogenic proteins such as PGC1α and UCP1 [183]. The latter data suggest that increased infiltration of immune cells in adipose tissue could affect the adaptive thermogenesis of BAT through a GIP-dependent mechanism. More recently, new findings suggest that the stimulation of GIPR could promote BAT function and thermogenesis, as well as the beigeing process, in human WAT precursors, in association with the amelioration of lipid metabolism [184]. These responses appear to require PKA activation [184], which is also involved in β3-adrenergic-mediated thermogenesis, as previously discussed [65]. Considering the favourable metabolic effects of both GLP-1 and GIP, combined therapies with both these incretins have been investigated to improve the efficacy of obesity treatments.

#### In vivo animal models studies

Multi-receptor pharmacology is at the forefront of next-generation therapies for the treatment of metabolic diseases. Indeed, the synergic effect of GLP-1 and GIP has already been investigated in vivo with the intra-cerebroventricular co-administration of both incretin hormones that significantly decreased body weight by reducing food intake through the central activation of anorexigenic POMC neurons located in the ARN of the hypothalamus [185]. Nevertheless, this weight-lowering effect was lost when either peptide was infused alone, suggesting that simultaneous central activation of GLP-1R and GIPR is essential for regulating energy balance [185]. It is already known that the ARN plays a critical role in metabolic regulation through a direct excitatory glutamatergic projection to the paraventricular nucleus (PVN) of the hypothalamus [186], which in turn modulates BAT activation and thermogenesis through sympathetic activity [187]. Considering the promising results obtained with combined infusion of GLP-1 and GIP peptides, unimolecular GLP-1/GIP receptor dual agonists were recently developed to promote different beneficial effects with administration of a single drug. Among these, NNC0090-2746 and tirzepatide showed improved efficacy in terms of EE, insulin sensitivity and weight loss, as compared to single GLP-1R agonism in a model of diet-induced obesity [188, 189]. Currently, it is not fully clear whether these beneficial effects are promoted by GLP-1R, GIPR or by simultaneous and possibly unbalanced activation of these receptors. Despite losing equivalent body weight, obese mice treated with tirzepatide showed a greater amelioration of systemic insulin sensitivity when compared to obese mice treated with GLP-1R monoagonist (semaglutide), an effect mediated by enhanced glucose disposal in both WAT and BAT [190]. The weight-independent insulin sensitization induced by tirzepatide appeared to be due to the engagement of GIPR. Indeed, similarly to long-acting GIPR agonist (LAGIPRA), tirzepatide favoured similar protection from obesity and insulin resistance, together with increased catabolism of glucose, lipids, and branched-chain amino acids (BCAA) in BAT. However, both compounds differed in differential regulation of > 1000 genes within BAT [190]. Thereafter, in a murine model of diet-induced obesity using stable-isotope tracers, it has been recently shown that tirzepatide stimulates catabolism of BCAAs/keto (BCKAs) acids in BAT, resulting in enhanced release of several intermediates of tricarboxylic acid (TCA) cycle (α-ketoglutarate, fumarate, and malate) and multiple amino acids (i.e. glutamate, alanine, etc.) principally involved in cold-induced thermogenesis [191]. Altogether, these data define, for the first time, the critical role of GIPR activation in mediating the metabolic outcomes of tirzepatide regardless of its weight-lowering action, highlighting an effect that involves the activation of a thermogenic-like amino acid signature in BAT.

#### Human studies

The clinical relevance of both NNC0090-2746 and tirzepatide was also investigated in clinical studies. While no significant improvement between NNC0090-2746 and liraglutide in terms of reduction of hyperglycaemia and weight loss was observed in patients with T2D [192], tirzepatide showed greater benefits on both these endpoints compared to semaglutide [193], thus becoming the most effective anti-obesity drug currently available [194]. In recent clinical trials, weekly administration of tirzepatide (15 mg) for 72 weeks has shown substantial and sustained reductions in body weight (> 25%) in 36.2% of obese patients recruited [195], and remarkable glycaemic benefits when compared to insulin degludec in patients with T2D [196]; similar results were achieved previously only by bariatric surgery, the first strategy with high remission rate of both obesity and T2D, but with several post-intervention complications [197].

Differences between these dual agonists could be explained by considering their affinity for the GLP-1R and GIPR. NNC0090-2746 equally binds both GLP-1R and GIPR, whereas tirzepatide acts as an unbalanced agonist showing higher potency for GIPR rather than for GLP-1R [192, 198]. The partial agonism of tirzepatide could be linked to its ability, when compared to native GLP-1, to induce less β-arrestin recruitment, a key signalling protein mediating GLP-1R internalization, thus resulting in higher cell-surface GLP-1R levels and enhanced beta-cellular responses in terms of insulin secretion [198]. These findings could potentially explain the superior efficacy of tirzepatide versus other balanced GLP-1R/GIPR dual agonists, even though it is not known whether this mechanism also affects the thermogenic capabilities of tirzepatide in humans. Although EE was not evaluated in the latest clinical trials, a study measuring food intake and calorie consumption in overweight patients using tirzepatide is currently ongoing [199]. In accordance with data obtained by studies in rodents [190, 191], Pirro et al. observed that tirzepatide reduced circulating BCAA in patients with T2D, and this effect was strongly associated with biomarkers indicative of enhanced insulin sensitivity [200]. Based on these preliminary results, additional investigation will be needed to establish whether the effects of GLP-1R/GIPR dual agonism on weight, glucose and lipid control are also a consequence of their ability to increase EE.

### GLP-1R/glucagon receptor dual agonists

Glucagon is a well-known peptide secreted by pancreatic α-cells that can correct hypoglycaemia by increasing hepatic glucose production [54]. Glucagon also exhibits pleiotropic properties, such as regulation of feeding behaviour (i.e. satiety and fullness) [201], lipid homeostasis [202], insulin secretion, and EE [203]. Glucagon acts mainly through a G-coupled receptor (GCGR) whose expression was found in hepatocytes, where this hormone displays principal functions in terms of stimulation of both gluconeogenesis and glycogenolysis [204]. GCGR was also identified in peripheral tissues (i.e. kidney, heart, spleen, thymus, stomach, duodenum, brain) [205] and its transcript has recently been reported in adipose tissue, particularly in mature adipocytes from both WAT and BAT samples [206]. GCGR distribution and activity in adipose tissue was established several years ago demonstrating the presence of high-affinity glucagon-binding sites in the soluble membranes of human fat cells [138]; in this setting, glucagon exhibited direct lipolytic action in a concentration range of 10^–6^–10^–8^ M [207] through a cAMP-dependent mechanism [208].

An effect of glucagon on thermogenesis was described several decades ago [209, 210], showing a decrease in body weight with an additional increase of EE following subcutaneous injection of glucagon in obese rats [211]. These effects seem to involve a BAT-dependent mechanism, since increases in temperature [212], blood flow, and cold-induced BAT mass [55] were observed following glucagon-based therapy. However, consistent data have indicated that glucagon could also affect heat production and BAT metabolism through indirect mechanisms. Relevant to this concept, a recent study in humans reported that individuals with active BAT (measured by FGD-PET) responded to glucagon infusion with higher rates of EE (230 kcal/day), similarly to cold activation but without any changes in BAT activity [213]. Of note, the role of glucagon in the regulation of thermogenesis has been better clarified in mice with genetic deletion of GCGR, in which a blunted cold-induced activation of adaptive thermogenesis and browning together with the reduction of PKA substrates phosphorylation in WAT was observed [31]. Based on these considerations, glucagon could be a potential molecule to counteract obesity through the activation of EE and BAT, even though the well-known hyperglycaemic excursions associated with this hormone [214] make it potentially harmful if used as a monotherapy for obese subjects with impaired glucose regulation. Therefore, combined therapies with hypoglycaemic agents, such as incretin mimetics, have been further explored.

#### In vivo animal models studies

The co-agonist ‘Aib2 C24 lactam 40k’, a chimeric peptide with higher selectivity for the GLP-1R and lower agonism for GCGR, was recently obtained through the chemical insertion of GLP-1 amino acid residues into the protein backbone of glucagon [215]. This peptide harbours modifications that allow it to maximize stability and efficacy in terms of resistance to cleavage by DPP4 and increased half-life, with a retained greater affinity for GCGR [215]. The enhanced efficacy of this new glucagon/GLP-1 chimeric peptide was assessed in DIO mice, in which weekly subcutaneous injections of this peptide for 1 month led to decreased body weight already in low doses mainly due to a loss of fat mass as compared to GLP-1-based therapy alone [215]. In the same study, O_2_ consumption and CO_2_ production were measured through indirect calorimetry and revealed an increased EE in mice treated by the dual agonist, suggesting that a metabolic shift towards heat production could contribute to the pronounced weight-lowering effect [215]. The mice did not exhibit changes in physical activity or food intake when on dual agonist therapy compared with untreated littermates, suggesting that the increased EE occurred mainly through increased basal metabolism and thermogenesis [215]. Interestingly, the dual agonist-induced fat loss was observed in visceral depots in which adipocytes acquired a small phenotype in association with increased phosphorylation of HSL suggesting that enhanced lipolysis could have a role in fat reduction [215]. The key component that primarily triggers fat burn is the glucagon-related sequence within the dual agonist peptide, since this hormone is known to enhance lipolysis, as reported by previous studies in which glucagon administration in human adipocytes induced a dose-dependent increase in lipolysis rates [138, 207, 216].

The synergistic contributions of combined GLP-1R and GCGR activation were also evaluated in GLP-1R-null mice maintained on an HFD, in which the dual agonist led to reduced body weight and adiposity due to an anorexic effect while hyperglycaemia was retained; this highlights that a GLP-1 fraction inside the chimeric compound is needed to protect against glucagon-induced hyperglycaemia [215]. Similarly, previous results have highlighted that therapy based on long-acting GLP-1R/GCGR dual agonist, named DualAG, exerted cumulative effects on food intake in rodents, thus reverting the obesity condition together with an amelioration of glucose tolerance [217]; however, these effects were ablated when both GLP-1R and GCGR were genetically eliminated [217]. Similar findings were observed after treatment with cotadutide, another dual agonist of both GLP-1R and GCGR, whose administration increased EE in DIO mice, with greater weight loss and a similar glucose-lowering effect compared with liraglutide [218]. The weight-lowering effects of cotadutide was also replicated in non-human primates, indicating that this drug maintains similar benefits in different species [218]. Furthermore, cotadutide produced body weight loss, amelioration of glucose tolerance, reduced liver fat content, and increased *mRNA* expression of the brown thermogenic genes *Ucp1*, *Pgc1α*, and *β3-AR* in an obese mouse model with leptin deficiency [219]. These beneficial responses were not sustained with single-agonist administration, suggesting that a specific mechanism involved in BAT activation was induced only when both GLP-1R and GCGR signalling were chronically activated [219]. Considering these results in preclinical models, the simultaneous activation of GLP-1R and GCGR could be a promising strategy to improve both adiposity and lipid profile without a worsening of glucose levels. In this regard, when GLP-1R signalling was abrogated, hyperglycaemic excursions were observed, indicating that GLP-1R activation is essential in offsetting glucagon-induced hyperglycaemia.

#### Human studies

The synergic effects of GLP-1 and glucagon were also explored in studies in human healthy volunteers in which combined infusion of low doses of both hormones increased EE to a greater extent than what was achieved with either peptide infused alone [220]. Based on these findings, development of unimolecular dual GLP-1R/GCGR agonists has been carried out to obtain new and improved anti-obesity drugs. The first of this new class of anti-diabetic agents tested in humans was oxyntomodulin, a 37-amino acid hormone secreted by the fundic cells of the colon [221]. When administered to obese subjects, this peptide successfully reduced calorie intake and increased EE, resulting in negative energy balance and significant weight loss [221]. These promising results encouraged the development of more suitable synthetic dual agonists, such as cotadutide and the more recent mazdutide, which successfully reduced body weight in exploratory analysis of obese and T2D patients [222, 223]. Noteworthy, the degree of body weight reduction with matzutide was similar to what was observed with tirzepatide (approximately 10% of body weight) after 12 weeks of treatment in patients with T2D and obesity, even though these data are referred to different study populations [195, 223]. Additional studies with a prolonged treatment period will establish whether this dual GLP-1R/GCGR agonist could reach the results obtained by tirzepatide [224]. Despite in vivo studies showed increased thermogenesis and BAT activity when dual GLP-1R/GCGR agonists were administered, currently no data regarding these outcomes are available in humans.

### GLP-1R/GIPR/GCGR triple agonists

#### In vivo animal models studies

The therapeutic spectrum of T2D and obesity therapy has recently been enriched by hybrid peptides acting as triple GLP-1R/GIPR/GCGR agonists that target both incretin and glucagon signalling to synergistically elicit favourable metabolic effects [225, 226]. The metabolic benefits of these triple agonists were found to be higher than mono and dual therapy [225, 226]. When obese mice were exposed to a triple GLP-1R/GIPR/GCGR agonist, a greater body weight loss was observed as compared with mice treated with GLP-1R/GCGR or GIPR/GCGR co-agonist or GLP-1RA monotherapy [226]. Furthermore, triple agonist treatment enhanced EE, likely through glucagon-mediated thermogenic effects [226]. It is reasonable to hypothesize that at least part of the signalling activity involved in these metabolic enhancements is derived from adipose tissue, as cAMP production was shown to be significantly stimulated in 3T3-L1-differentiated adipocytes exposed to the triple agonist [226]. Further experiments were conducted in mice with deletion of GLP-1R, GIPR, and GCGR to explore the synergistic involvement of these receptors in the metabolic responses. A blunted weight-lowering effect, particularly in obese GLP-1R- and GCGR-null mice, was observed, as these receptors were no longer able to signal for enhanced anorectic actions [226]. However, when GIPR was genetically abrogated, glucagon-dependent hyperglycaemic excursions were enhanced during triple agonist therapy, highlighting that GIPR signalling functions for the maintenance of glucose balance [226]. These findings suggest that synergic activation of GIPR, GLP-1R, and GCGR could clinically ameliorate body weight by potentiating thermogenic responses that do not occur when only a single receptor is activated. The triagonists MAR423, HM15211 and LY3437943 were recently developed and are being assessed in early clinical and preclinical trials. Animal studies confirmed the weight-lowering efficacy, increased energy consumption, and lipid profile improvement in models of obesity and metabolic abnormalities, highlighting that these novel compounds could have thermogenic potential [47, 48, 225–227]. Particularly, recent studies showed that HM15136 promoted WAT browning in obese mice by increasing *mRNA* expression of *Pgc1α* and *Ucp1*, leading to enhanced EE [228].

#### Human studies

To date, the clinical relevance of triple GLP-1R/GIPR/GCGR agonists has not been fully established. The HM15211 compound is being tested in phase 1 clinical trials for obesity and non-alcoholic steatohepatitis treatment [229], while the clinical efficacy of MAR423 has not yet been studied. Currently, LY3437943 is the triple agonist in the most advanced stage of clinical assessment, showing promising results in a recent phase 1b trial with robust reductions in glucose and weight to a similar extent as tirzepatide and mazdutide [230]. However, key of thermogenic responses such as EE have not yet been assessed. No consistent data regarding the browning or BAT activation by triple agonists are available from clinical trials and further human studies are needed in this regard.

## Conclusions and future perspectives

Appetite inhibitors and inhibitors of the absorption of nutrients represent the pharmacological agents currently recommended for the treatment of obesity. However, the control of body weight under these therapies is often not achieved and/or maintained in the long-term due to counter-regulatory mechanisms modulating energy disposal and thermogenesis. In recent decades, the rediscovery of metabolically active BAT in adulthood provoked strong interest in the identification of new browning molecules to counteract obesity and related metabolic complications (Fig. 1 and Table 1), including emerging poly-agonists, with potential for induction of thermogenesis and browning. The recent clinical results achieved with the selective β3-AR agonist mirabegron have shown improvements in multiple measures of glucose metabolism in obese and insulin-resistant individuals probably driven by activation of the beigeing process. Similarly, preclinical results described TRβ and FXR agonists as good candidates for obesity treatment through the activation of thermogenic and browning responses occurring via direct or indirect mechanisms.

In addition, several preclinical and clinical studies have demonstrated that novel incretin and glucagon mimetic compounds initially developed for the treatment of T2D, such as the dual agonists GIPR/GLP-1R and GLP-1R/GCGR and the triple agonist GLP-1R/GIPR/GCGR, can enhance BAT activity and browning of WAT, introducing a new perspective for the management of obesity. The pharmacological activation of GLP-1R, GIPR, GCGR triggers several intracellular mediators (p38 MAPK, PI3K/AKT, AMPK/SIRT1/PGC1α) whose combined activation may ultimately enhance BAT activity and browning through the upregulation of UCP1, the key transducer of thermogenesis (Figs. 1 and 2). Therefore, these novel molecules, differently from older anti-obesity drugs (e.g. orlistat), could potentially lead to effective weight management in the long-term through the sustained activation of thermogenesis, thereby reducing the risk for weight-loss resistance and weight regain. Further studies are needed to elucidate whether thermogenic and browning responses associated with these agents are directly elicited on adipocytes or are achieved by indirect mechanisms, potentially involving the CNS.

## Data Availability

A data availability statement is not applicable since this manuscript reviews current literature regarding thermogenesis and new clues of obesity pharmacotherapy.
